# A hybrid approach with metaheuristic optimization and random forest in improving heart disease prediction

**DOI:** 10.1038/s41598-024-73867-x

**Published:** 2025-03-31

**Authors:** Geetha Narasimhan, Akila Victor

**Affiliations:** School of Computer Science and Engineering, Vellore Institute of Technology, Vellore, Tamilnadu India

**Keywords:** Metaheuristic, Feature selection, Optimization techniques, Genetic algorithm (GAO), Particle swarm optimization (PSO), Ant colony optimization (ACO), Random Forest (RF), Cardiology, Health care, Data acquisition, Data integration, Data mining, Data processing, Machine learning, Software

## Abstract

Cardiovascular diseases (CVD)  a major cause of morbidity and mortality among the world’s non-communicable disease incidences. Though these practices are in use for diagnostics of different CVDs in clinical settings, need improvement because they are solving the purpose of only 57% of the patients in emergency. Due to this cost of diagnosis for heart disease is increasing which is the reason for analyzing heart disease and predicting it as early as possible. The main motive of this paper is to find an intelligent method for predicting disease effectively by means of machine learning (ML) and metaheuristic algorithms. Optimization techniques have the merit of handling non-linear complex problems. In this paper, an efficient ML model along with metaheuristic optimization techniques is evaluated for heart disease dataset to enhance the accuracy in predicting the disease. This will help to reduce the death rate due to the severity of heart disease. The SelectKBest feature selection is applied to the Cleveland Heart dataset and overall rank is obtained. Accuracy is measured. The optimization techniques namely Genetic Algorithm Optimized Random Forest (GAORF), Particle Swarm Optimized Random Forest (PSORF), and Ant Colony Optimized Random Forest (ACORF) are applied to the Cleveland dataset. Classification algorithms are performed before and after optimization. The output of the experiment explains that the GAORF performed better for the dataset considered. Also, a comparison is made along with the SelectKBest filter methods. The proposed model achieved better accuracy which is the maximum among other optimization and classification techniques.

## Introduction

Healthcare Challenges are misdiagnosis and delayed diagnosis are prevalent, impacting 5–15% of patients globally. Conventional methods miss early detection and struggle with complex diseases. Machine Learning to the rescue offers improved accuracy and handles large data volumes for better diagnosis. ML can predict and prevent diseases, potentially reducing mortality. CVD leading cause of death worldwide, responsible for approximately 17.9 million fatalities each year^1–5^. Current methods like ECGs and echocardiograms only accurately diagnose 57% of CVD patients in emergencies. Many CVD patients are asymptomatic or have undetectable changes, making early diagnosis crucial. The solution would be integrating medical algorithms with existing techniques that improve accuracy and facilitate better decision-making in diagnosis, treatment, and research. Leverages patient data for disease identification, risk prediction, and refined diagnoses^6–10^.

The challenges to using medical algorithms are lack of awareness, uncertain capabilities, complex results, and access issues that hinder adoption. Automation could improve accuracy, and data sharing, and reduce administrative burden. The methodology used is Data Acquisition to collect patient data (age, weight, blood pressure, etc.). Feature Selection method is used select relevant features relevant features. Optimization Algorithms like Genetic Algorithm: Evolves solutions like natural selection. Particle Swarm Optimization: Particles learn from each other to find the best solutions. Ant Colony Optimization: Pheromone trails guide “ants” to the best solutions. Performance Evaluation to measure classifier accuracy on unseen data. Comparison to select the most accurate algorithm for the dataset. The results are the optimized features for heart disease classification and the potential for diagnostic tools and improved treatments. The model uses the Cleveland Dataset (303 records, 13 features). Compared 3 optimization algorithms (PSO, ACO, Genetic). Random Forest was chosen as the best-fitting ML algorithm.

The work is approached with a Hybrid model that uses the PSO for optimizing the local solution ACO is adopted for continuous optimization of the local solution. This adaptation reduces the complexity and of complex datasets accuracy is improved. The rank of the selected system is obtained using the SelectKBest filter feature and thus fed to classification algorithms. When it comes to classification algorithms Support Vector Machine (SVM), Random Forest (RF), Naïve Bayes(NB), Logistic Regression(LR), Decision tree(DT), Random Forest with Grid Search (RFGS) and Extended Gradient Boost (XGB)^12^. By optimizing the data with GAO, PSO, and ACO which is fed to RF to obtain the best possible accuracies with improved performances. In this study, the feature selection with three different techniques and an overall ranking method is applied rather than relying on a single technique. Also, three different optimization techniques are applied to find the best method for the dataset and adopted with machine learning techniques. The study suggested that GAO with RF performs better for the dataset considered for the study. The list of symbols used in the study are listed in Table 1.

**Table 1:** List of symbols used in the manuscript and its explanation.

Symbol	Meaning	Description
ML	Machine Learning	A field within artificial intelligence and computer science that focuses on utilizing data and algorithms to create AI systems that mimic human behavior.
GAORF	Genetic Algorithm Optimized Randon Forest	A technique for addressing both constrained and unconstrained optimization problems by simulating the natural selection process inspired by biological evolution.
PSORF	Particle Swarm Optimized Random Forest	This is a population-based selection. This algorithm is to obtain global optimum fitness function in a given area.
ACORF	Ant Colony Optimized Random Forest	This is probabilistic method for solving computational problems which can be reduced to find good paths through graphs.
CVD	Cardiovascular diseases	Is a general term for conditions that affect heart or blood vessels.
RF	Random Forest	This algorithm combines multiple decision tree’s output to reach one result.
SVM	Support Vector Machine	This algorithm solves complex classifications, and other regression, outlier detection by carrying optimal data transformation.
NB	Naïve Bayes	This algorithm solves classifications where high dimensional datasets are trained.
LR	Logistic Regression	This algorithm performs binary classification by estimating the probability of a given outcome, event, or observation.
DT	Decision Tree	This algorithm is used to make predictions based on the decision trees.
RFGS	Random Forest Grid Search	This method is used to tune the hyperparameters of Random Forest algorithm in ML
XGB	Extended Gradient Boosting	A ML algorithm used to solve regression, classification, user defined predictions ranking problems.
KNN	K Nearest Neighbor	This algorithm determines classifications or predictions by analyzing the proximity of an individual data point to others.
UCI	University of California	A machine learning repository which consists of 665 datasets from various fields.

This article highlights the boundaries of current diagnostic approaches for heart disease, emphasizing the need for improvement. It emphasizes the importance of early prediction for better patient outcomes and reduced mortality rates. The model introduces the concept of using ML and metaheuristic algorithms for improved heart disease prediction. It showcases the potential of using optimization techniques like Genetic Algorithm (GAO), Particle Swarm Optimization (PSO), and Ant Colony Optimization (ACO) to enhance the accuracy of machine learning models like Random Forest. This methodology provides a specific example of using the SelectKBest feature selection technique on the Cleveland Heart Disease dataset. It demonstrates the process of comparing the performance of different optimization techniques (GAORF, PSORF, ACORF) with a baseline model (Random Forest). The proposed model establishes a benchmark for accuracy achieved using the proposed GAORF model on the Cleveland dataset. It encourages further research by mentioning the possibility of achieving even better accuracy with different techniques.

Our proposed work is categorized into 5 different sections “Related work” , which refers to the literature on ML algorithms for machine learning processes that detect CVDs, “Materials and methods” section, which discusses the framework for the detection of CVDs, design setup, and features selected and “Optimization algorithms” section, discusses different machine learning algorithms for optimization of a better algorithm for the dataset. In the final section, results extracted from different models are done with performance evaluation that decides the best algorithm from the work. In “Materials and methods” section, machine learning algorithms like the PSO, GA, ACO are applied to the selected datasets^44,45^.

### Need for hybrid ml model for diagnosis of cardiovascular diseases

Extensive literature is reported in predicting heart disease considering a single classification or combination of algorithms known as hybrid techniques^13^. Despite this very few researchers have focused on optimization techniques and however, researchers have failed to analyze the difference between these optimization techniques when applied for a single dataset.

**Fig. 1:**
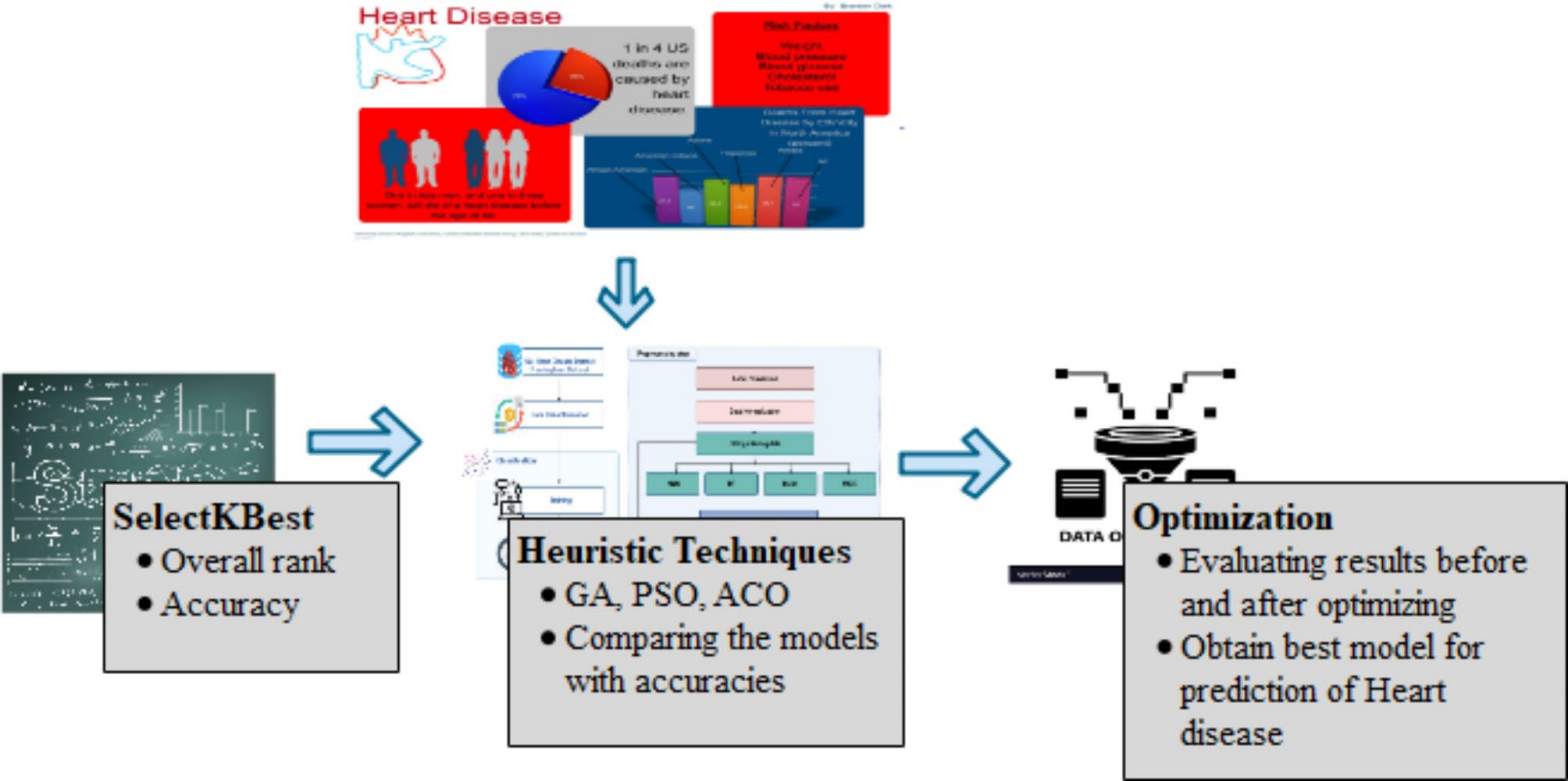
Optimization model for cardiovascular disease diagnosis^49–51^.

In this proposed framework, the SelectKBest Method is used to find the rank of each feature using Chi-square, Mutual information, and F-statistics. The overall rank is considered and based on that features are selected and given to classification techniques. GAO, ACO and PSO optimize the dataset and given to classification techniques is given to classification techniques. A comparison of classification algorithms is evaluated before and after optimization to determine an optimized model for the selected dataset that gives better accuracies where the workflow for the hybrid model is shown in Fig. 1.

## Related work

### Various situation

The objective is to predict heart disease by applying swarm intelligence and ANN for which researchers used UCI dataset and applied PSO algorithm for feature selection^6^. ANN is applied after features are selected and found PSO performed better in predicting heart disease. In the case of heart diseases, the prediction using PSO and ACO along with other ML techniques, the PSO using feed-forward has resulted in better accuracies^12^. A combined method of ML using Mutual information(MI) and Binary PSO referred to as MI_BPSO is used for optimization to predict heart disease^3^. It is found that random forest with integrated Ant colony particle swarm optimization works better and achieves better performance after the PSO. Similarly^14^ worked on heart disease prediction using optimization techniques. The PSO algorithm is combined with logistic regression for classifying heart disease binary, feature selection using dragonfly algorithm and found that have high accuracies^13,15,16^. In^17^, a model was created for predicting heart disease. The model used a binary artificial bee colony for finding the best features and KNN was used for the classification technique. The model achieved 92.4% accuracy.

Khourdifi, Y., & Bahaj, M in 2019 used PSO along with MLP for predicting heart disease^9^. The dataset is used from the UCI repository namely the Cleveland dataset. The model achieved around 84.61% accuracy, whereas in^18^ modified Artificial bee colony-dependent feature selection is applied for predicting heart disease. In^19^ an optimal feature subset selection based on ACO is used to predict breast cancer and liver disorder with the model achieving 96.56% and 92.44% respectively. In^20^ a cuckoo-inspired algorithm for feature selection for predicting heart disease. In this method, the Cuckoo search algorithm performed better than the Cuckoo optimization algorithm. Asadi et al., in 2021 used ACO for classifying data and the proposed model used swarm optimization for feature selection^21^. Velswamy K et al. used random forest along with multi-objective swarm optimization for predicting heart disease in 2021^22^.

Jabbar M.A, in 2013 used a model for the classification of heart disease and Modified bee algorithms for feature selection. SVM, Naïve Bayes, DT, and RF are used for classification^23^. Deekshatulu and Chandra. P in 2013 has also used Associative classification and genetic algorithms for predicting heart disease^24^. Another work of^25^ used KNN and genetic algorithms for the classification of heart disease. Bahassine et al., in 2020 developed a classification algorithm for predicting heart disease and for feature selection using a GA.

Gene expression datasets offer vast amounts of information for analyzing biological processes; however, the presence of redundant and irrelevant genes makes it challenging to identify key genes in high-dimensional data. To overcome this issue, several feature selection (FS) methods have been introduced. Enhancing the efficiency and accuracy of FS techniques is crucial for extracting significant genes from complex multidimensional biological data^46,47^. The authors created a new method called CSSMO that combines two algorithms to select a smaller set of important genes from the data. Using fewer genes allows machine learning algorithms to work faster. The selected genes should be informative for cancer prediction. The authors tested their method on eight datasets and found it to be more accurate than other existing methods. Overall, this research introduces a new technique for selecting genes that might be useful for building more accurate and efficient models for early cancer prediction^48^. This paper specifically looks at how machine learning algorithms are used with medical data to categorize different cancers and even predict their outcomes. The paper dives into supervised, unsupervised, and reinforcement learning, explaining their strengths and weaknesses. By accurately classifying cancers, predicting patient outcomes, and identifying potential treatment targets, machine learning has the potential to revolutionize cancer diagnosis and treatment. This review equips readers with the latest advancements in machine learning for cancer classification, allowing them to decide on its use in clinical settings. The paper also discusses the potential for even more powerful machine learning systems in the future. Based on the background study a model is proposed for predicting heart disease.

## Materials and methods

### Proposed methods

**Fig. 2:**
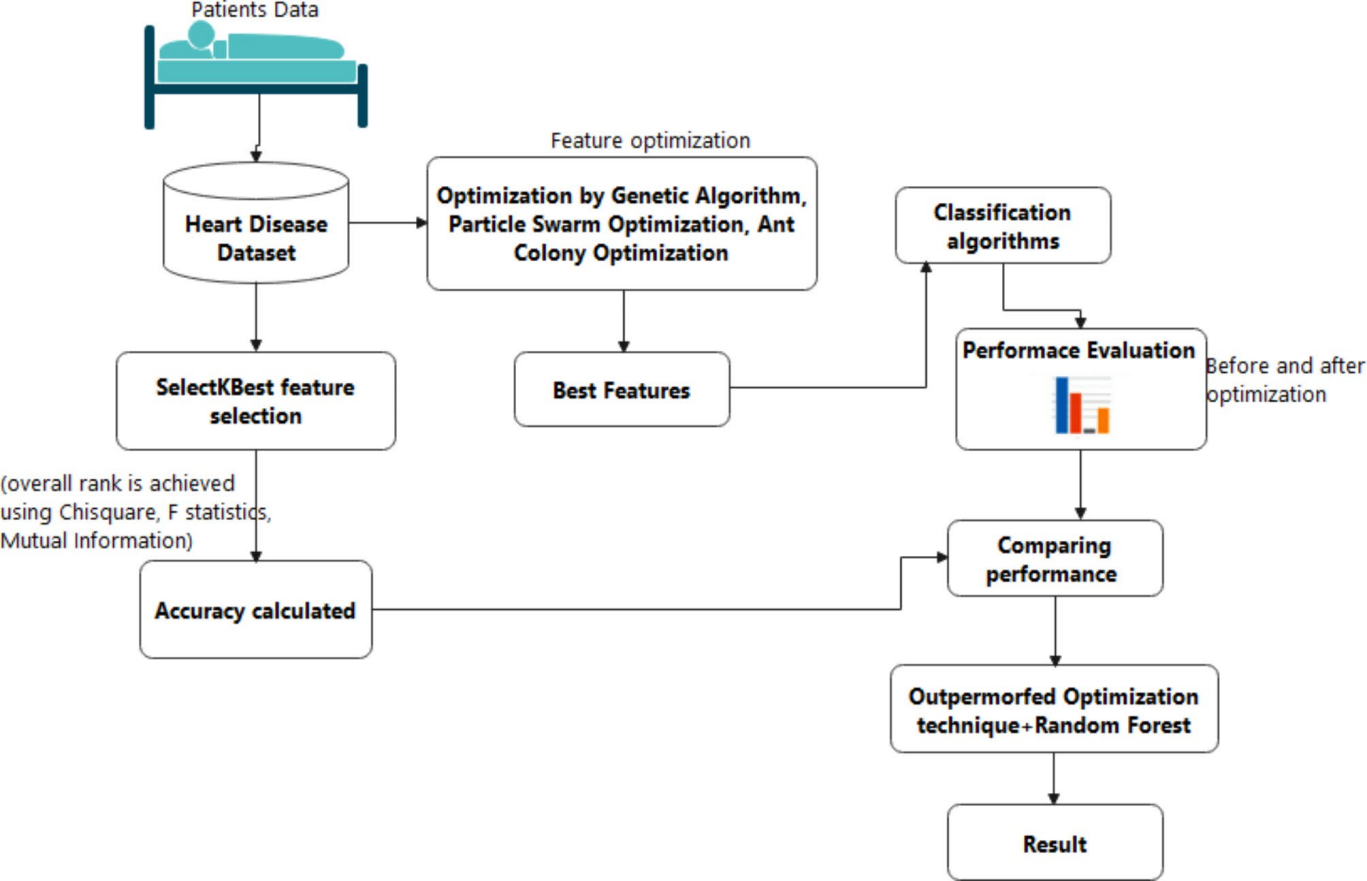
Workflow of the proposed model. The proposed model follows a structured process, starting with the dataset obtained from the UCI repository, which undergoes preprocessing. The refined dataset is then processed through GAO, PSO, and ACO algorithms for feature optimization. The optimized features are subsequently utilized by machine learning techniques, and the model’s performance is assessed.

The proposed model gives a detailed idea of the model and its behaviour. In this paper, the model proposed consists of gathering the dataset, preprocessing the dataset, applying optimization techniques, and evaluating the accuracy of the classification algorithms. The study utilizes the Cleveland dataset, which comprises 13 features and a single target class. The framework for the proposed model is given in Fig. 2. The dataset is preprocessed using the SelectKBest feature and election is applied where overall rank is obtained with the accuracies. Then the dataset will be given to optimization techniques namely GAO, PSO, ACO. These techniques are applied to the dataset and a subset of features are selected ignoring the unrelated features. The subset of features is then used by classification algorithms. The performance metrics are evaluated from which RF performed better before optimization. Therefore, RF along with a subset of features selected by each optimization is evaluated and accuracy is found. The higher accuracy model is selected and proved to be the best optimization technique for the selected dataset.

### Dataset preparation

The dataset is used for training ML model and metaheuristic algorithms the dataset is much needed. The dataset is available publically in repositories like UCI and Kaggle. The Cleveland dataset which has 303 records^10^ shown in Table 2. The Cleveland dataset comprises 13 independent variables and one target feature with 2 classes namely heart disease and no heart disease.

This dataset is considered the standard dataset for research purposes and it has very minimum missing values.

**Table 2:** Attributes of the heart dataset from uci with feature details ^38^.

Attribute	Description	Type	Value Range
Age in years	Continuous value	Numeric	29 < age > 77
Sex	1 represents male 0 represents female	Nominal	1-male 0-female
CP-Chest pain	The chest pain type is categorized as follows: 0 represents typical angina, 1 indicates atypical angina, 2 corresponds to non-anginal pain, and 3 signifies asymptomatic cases.	Nominal	4-asymptomatic 3-non anginal pain 2-atypical angina 1-typical angina
trestbps	Resting blood pressure	Numeric	> 160: very high 140–160: High 120–140: Unusual 90–120: Normal
chol	Serum cholesterol in mg/dl	Numeric	> 250: very high 110–200: Normal 200–240: borderline high 240–250: High
Fasting Blood sugar	Fasting blood sugar > 120 mg/dl 1	Nominal	1-true 0-false
Restecg—resting electrocardiographic (ECG)	resting electrocardiographic results are classified as follows: 0 indicates a normal result, 1 signifies the presence of ST-T wave abnormalities, and 2 suggests possible or definite left ventricular hypertrophy	Nominal	0-normal 1-STTwave abnormality 2-showing probable
thalach	heart rate achieved	Numeric	60-100-normal > 100- tachycardia
Exangexercise-induced angina	Blood supply when you exercise 1 represent yes 0 represents no	Nominal	1 0
Old peak	ST depression induced by exercise relative to rest	Numeric	0-6.2
Sl: slope	The slope of the peak exercise ST segment 0 represents upsloping 1 represents flat 2 represents downsloping	Nominal	1-upsloping 2-flat 3-downsloping
Ca	number of vessels (0–3) coloured by fluoroscopy	Nominal	0, 1, 2, 3
thal	Thallium stress test 1 represents normal; 2 represents fixed defect; 3 represents a reversible defect	Nominal	3-normal 6-fixed 7-reversible
target	The predicted attribute 0 represents no chances of heart failure 1 represents chances of heart failure	Class variable	0-no heart disease 1-heart disease

### Feature selection-ethical consideration

The initial step is finding the accuracy using SelectKBest feature selection which is obtained by applying the Chi-square, Mutual information, and F-statistics. The chi-square is used for categorical data. In statistics, the chi-square test is utilized for testing the event’s independence. The formula is given in Eq. (1)^27^

1 $$\:{\varSigma\:\chi\:}_{c}^{2}=\frac{({O}_{i}-{E}_{i}{)}^{2}}{{E}_{i}}$$

Where O is observed cases, E is the expected values, $\:{\chi\:}_{c}^{2}$ is Chi-square value Mutual information is the measurement of two random variables that calculates the information obtained about one variable through another random variable. The probability distribution function (p.d.f) of mutual information p (x, y) is calculated using Eq. (2)^27^. These values are integral parts of the feature but mentioning them here is to justify understanding that data for selected features fits into the distribution.

2 $$\:I\left(X;Y\right)={\int\:}_{X}{\int\:}_{Y}p\left(x,y\right)log\frac{p(x,y)}{p\left(x\right)p\left(y\right)}dxdy$$

Where p (x, y) denotes the joint probability density function, the marginal density function is given by p(x) and p(y). The F-statistics is the value obtained after conducting the ANOVA test which is the mean of two populations. The F-statistics are given in Eqs. (3) (4) (5)^28,29^.

3 $$\:\text{F}=\frac{Sum\:of\:squares\:between\:groups/df1}{sum\:of\:squares\:within\:groups/df2}$$

This study utilizes the SelectKBest method, which picks the top K features based on their scores calculated using three different functions: Chi-squared: Analyzes the dependence between a feature and the target variable. Higher scores indicate stronger relationships. F-statistic: Compares the variance between groups defined by the target variable. Higher scores suggest features that better differentiate groups. Mutual information: Measures the shared information between a feature and the target variable^52^. Higher scores imply features that provide more information about the target. Each feature receives a rank based on its score for each method. The top-ranked feature gets a rank of 1, and so on. These individual ranks are then combined to generate an overall rank for each feature, indicating its collective importance across all scoring methods. The results of feature selection are shown in Tables 3, 4 and 5 respectively. These tables explain the features alongside their corresponding scores, aiding in understanding which features contribute most to accurate heart disease prediction.

## Optimization algorithms

### Genetic algorithm (GAO)

**Fig. 3:**
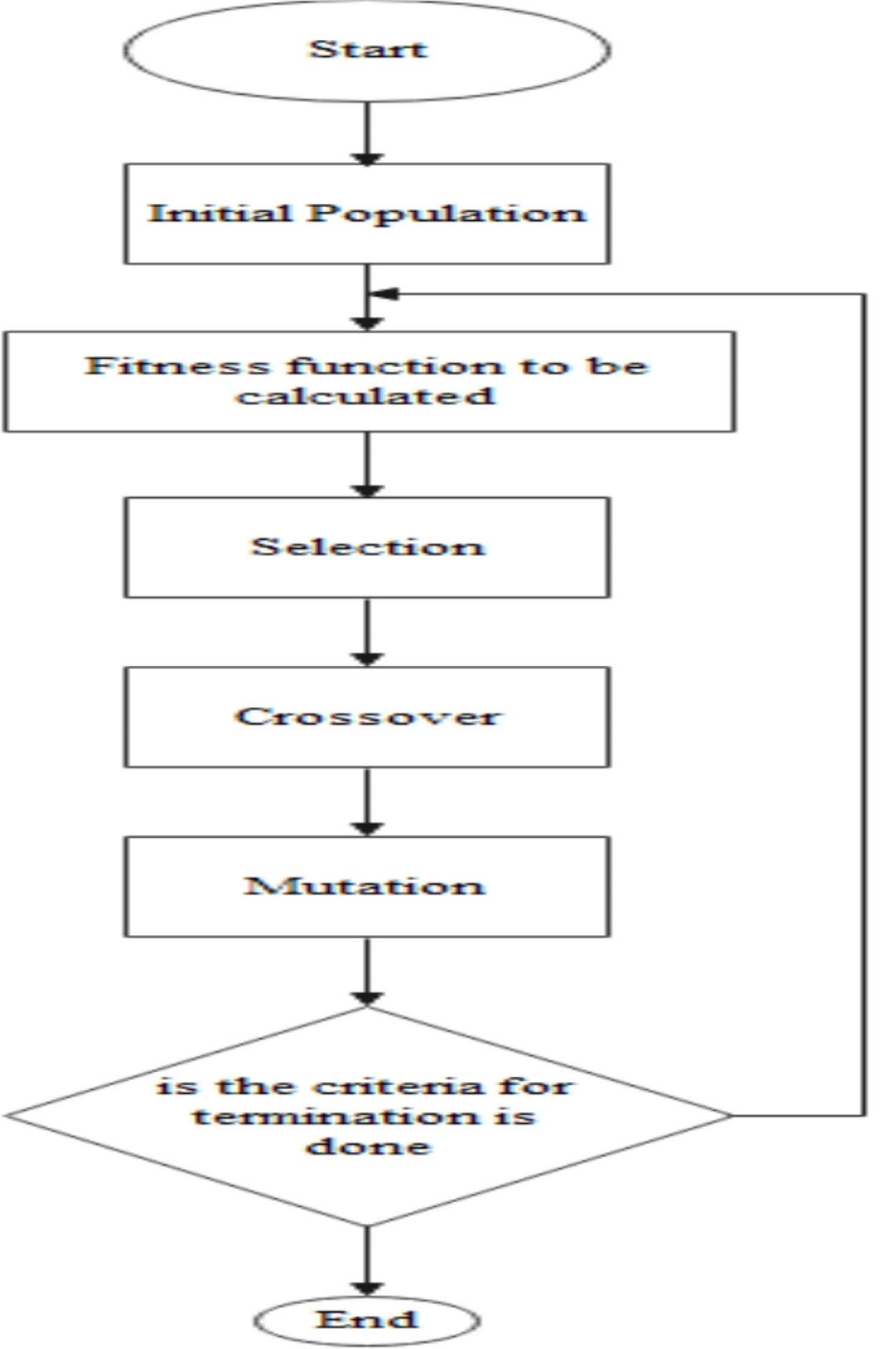
Genetic algorithm flowchart^31^.

GA is mainly used for selecting the feature subset from the given dataset. This method consisted of terms like selection, chromosome, genes, individual, nutation, crossover, and fitness function. This method was developed by Goldberg in 1989^30^ and the method is used to generate a population of fixed size called search space. The entities from the population are selected randomly, combined, and mutated, later they can be ignored are selected based on their fitness levels. To create the next level generation, the selection operation is performed. A combination of genes is called a chromosome. DNA blocks are genes. The population is the individuals with the same chromosome length, and the value assigned to them is fitness^32^. The flow of GAO is given in Fig. 3. The fitness function is denoted by Eqs. (4),

4 $$\:\text{f}\left(\text{x}\right)=\frac{1}{1+g\left(x\right)}$$

the objective function given by g(x) and the fitness function given by f(x).

The value assigned to the individual is carried by fitness function f(x). Genes from parents combine to form new chromosomes in a crossover, randomly changing the gene is a mutation, and creating the next generation is selection. Algorithm 1 presents the pseudocode for GAO.

**Algorithm 1:**
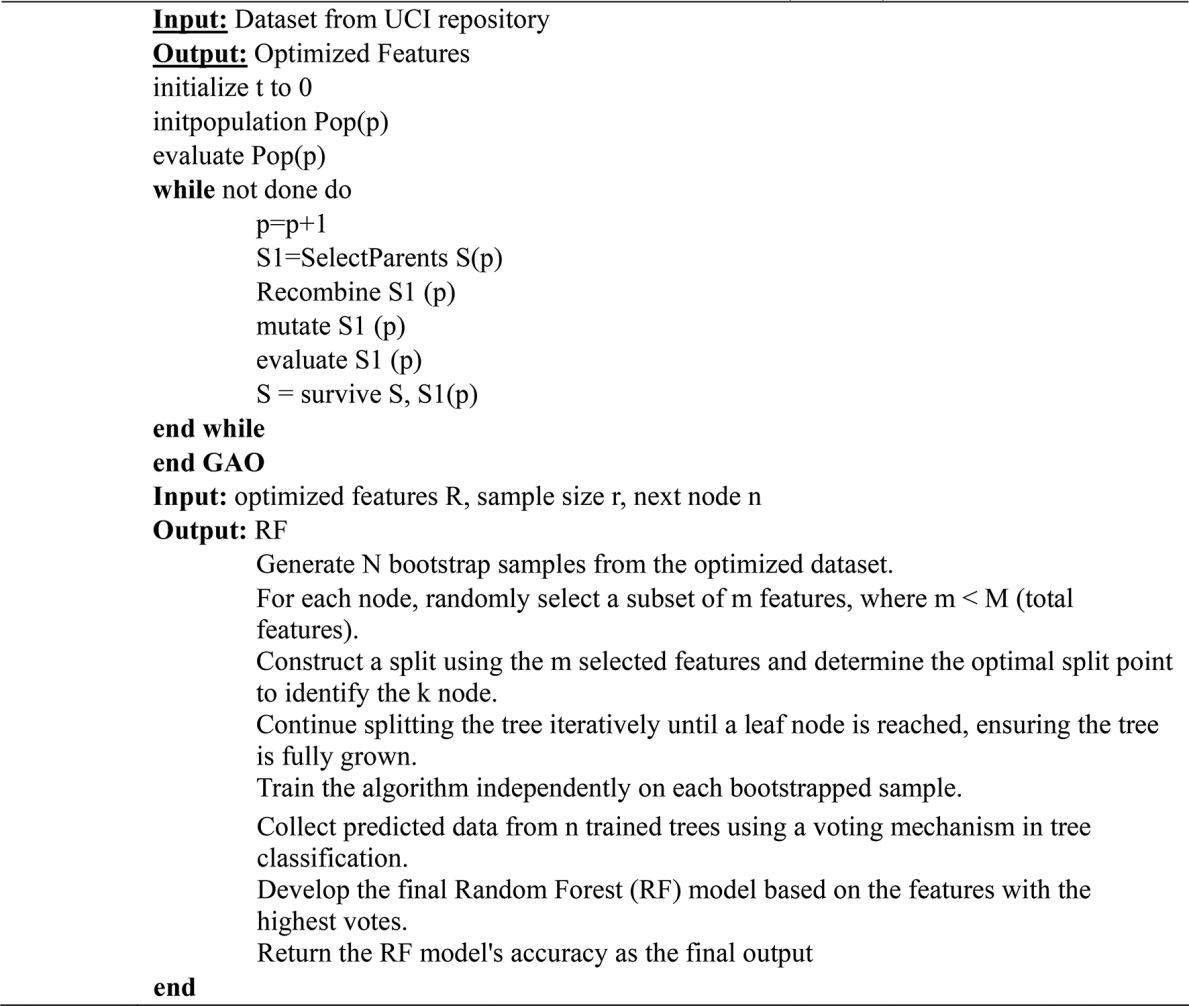
Genetic algorithm optimization–Random Forest (GAORF).

### Ant colony optimization (ACO)

**Fig. 4:**
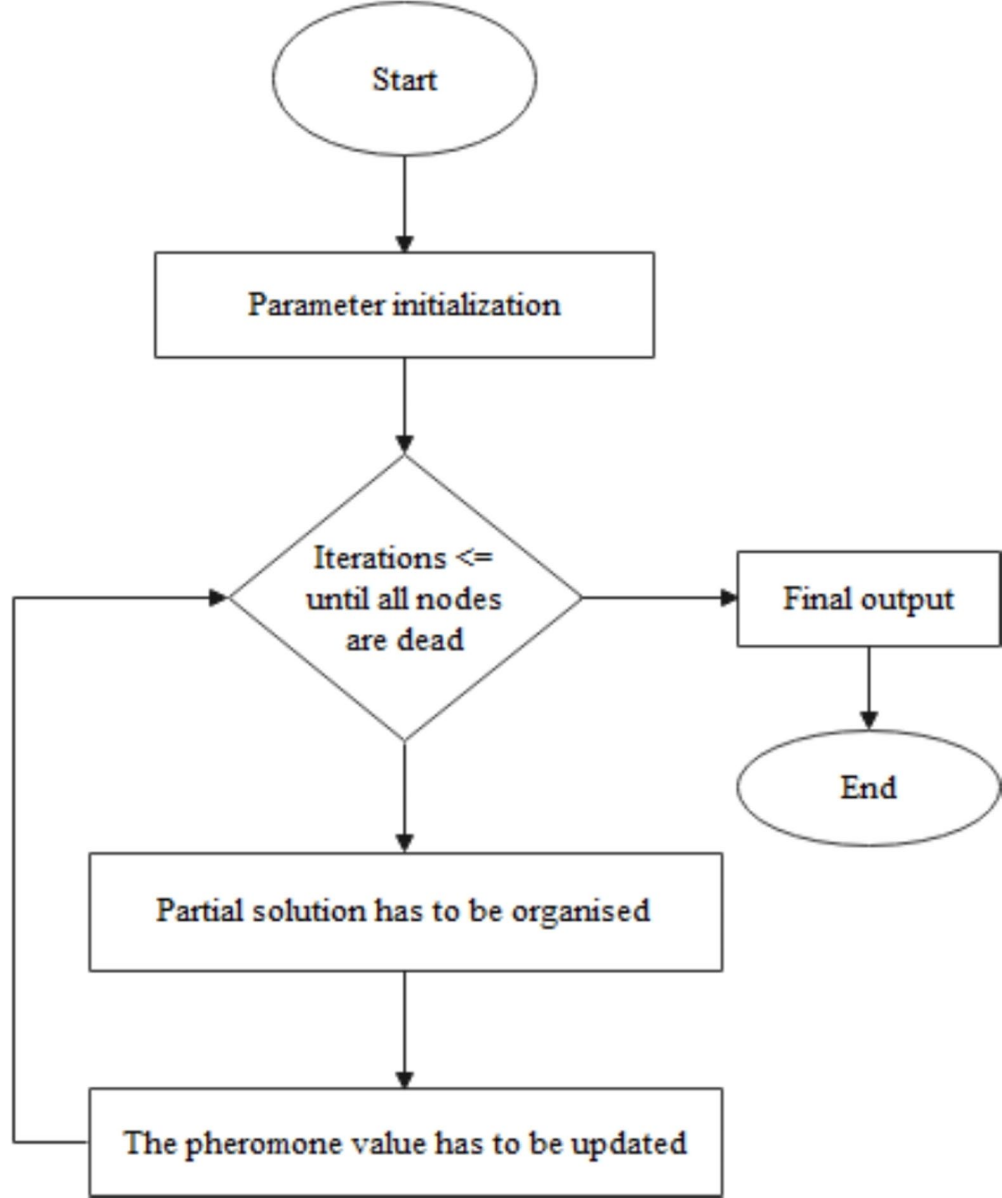
Ant colony optimization flowchart^11^.

This section explains the feature selection procedure carried out by Ant Colony Optimization. The flowchart of the ACO is shown in Fig. 4. In this model, the features of the dataset are independent nodes. Based on the probability of the selection p _k_(i), features are selected^33^. Eq. (5) gives the probability of selection.

5 $$\:{P}_{k}\left(i\right)=\frac{\left[\tau\:\right(i\left){]}^{\alpha\:}\:\right[\eta\:\left(i\right){]}^{\beta\:}}{{\varSigma\:}_{l\in\:{N}_{i\:to\:k}}\left[\tau\:\right(l\left){]}^{\alpha\:}\right[\eta\:\left(i\right){]}^{\beta\:}}$$

Where $\:\eta\:\left(i\right)$ is feature frequency and it denotes the number of features in the training and it also denotes the heuristic information that the ants have. The feasible neighbourhood of ants is denoted by $\:{N}_{i\:to\:k}$. The feature i pheromone trail value is mentioned by $\:\tau\:\left(i\right)$. ACO^34^ parameters are initialized as $\:\alpha\:\:and\:\beta\:$. The trial of pheromone is updated based on the global update rule and mentioned in Eq. (6).

6 $$\:\tau\:\left(i\right)=\rho\:\tau\:\left(i\right)+\sum\:_{k=1}^{n}\varDelta\:\tau\:\left(i\right)\left(i\right)$$

$\:{\uprho\:}$ denotes the evaporation parameter that decays the pheromone trail and the number of ants is denoted by $\:\eta.$ The proposed ACO works for feature selection. Features have similar probability selection $\:{P}_{k}\left(i\right)$. The roulette wheel selection algorithm^35^ is used for selecting n features.

This method is more suitable for feature selection problems, as this method has no heuristic that guides the optimal search all the time. Considering the graph, ants will identify the best combinations of features^11^. The pseudocode for the ACO is mentioned in Algorithm 2.

**Algorithm 2:**
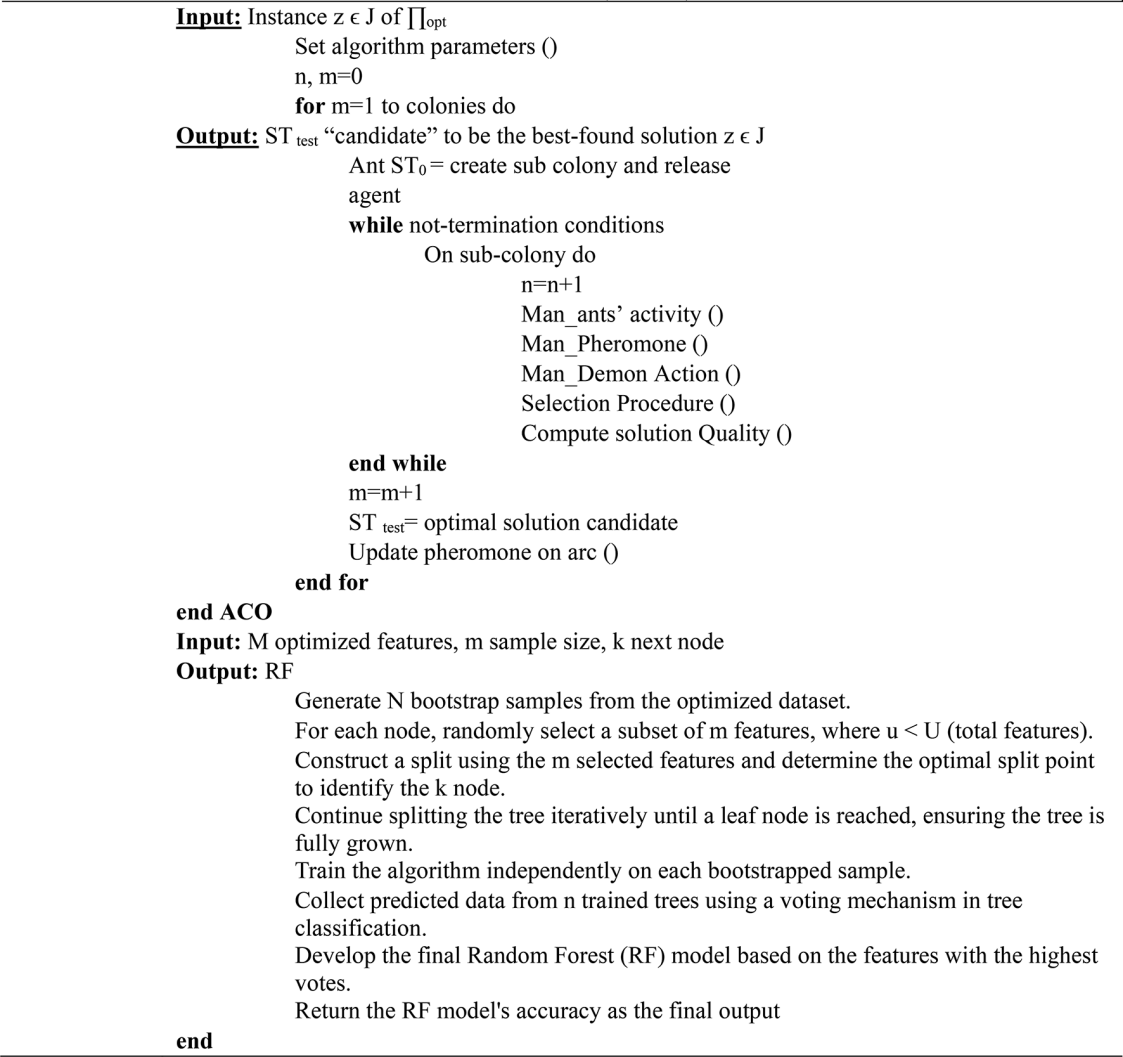
Pseudocode for Ant Colony Optimization—(ACORF).

### Particle swarm optimization (PSO)

Solving the complex problem by interacting between simple agents and their environment is carried out by particle swarm optimization. Russel 1995 developed this algorithm from the inspiration of a metaheuristic setup. Each article keeps moving at every iteration.

**Fig. 5:**
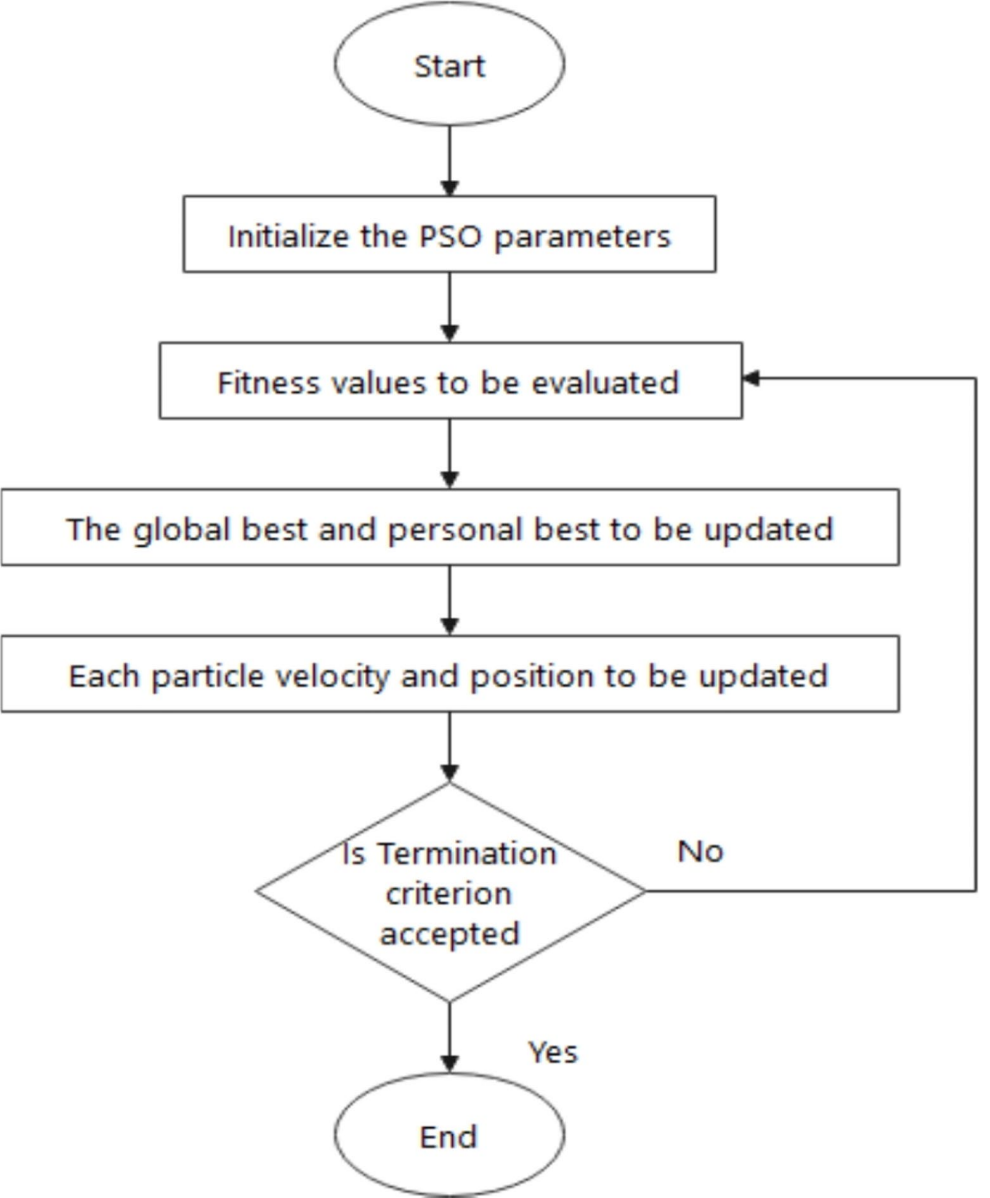
Particle swarm optimization flowchart^11^.

The PSO has two equations, the velocity update and particle position. Based on dimensions the fitness value is calculated^37^. The updated equation and position are given below in Eq. (7), and (8).

7 $$\:{v}_{id}^{t+1}={w}_{i}{v}_{id}^{t}+{c}_{1\:}rand\left(\right)\:({p}_{id}^{t}-{x}_{id}^{t})+{c}_{2}\:rand\left(\right)\:({p}_{nd}^{t}-{x}_{id}^{t})$$

8 $$\:{x}_{id}^{t+1}={x}_{id}^{t}+{v}_{id}^{t+1}$$

Here w is inertia weight, $\:{c}_{1\:}and\:{c}_{2}$ are acceleration constants, a random function denoted by rand (). The personal best at *t*th iteration is given by Eq. (9)

9 $$\:{P}_{i}^{t}=[{P}_{i1}^{t},{P}_{i2}^{t},\ldots {P}_{id}^{t},\ldots {P}_{im}^{t}]$$

and local best is given by Eq. (10)

10 $$\:{P}_{n}^{t}=[{P}_{n1}^{t},{P}_{n2}^{t},\ldots{P}_{nd}^{t},\ldots {P}_{nm}^{t}]$$

The one particle close to the optimum, informs its position to get modified. The most effective is the extension of combinatorial optimization. The main code of this algorithm is to move the particle to find the optimum^36^. The pseudocode for PSO is given in Algorithm 3^37^.

**Algorithm 3:**
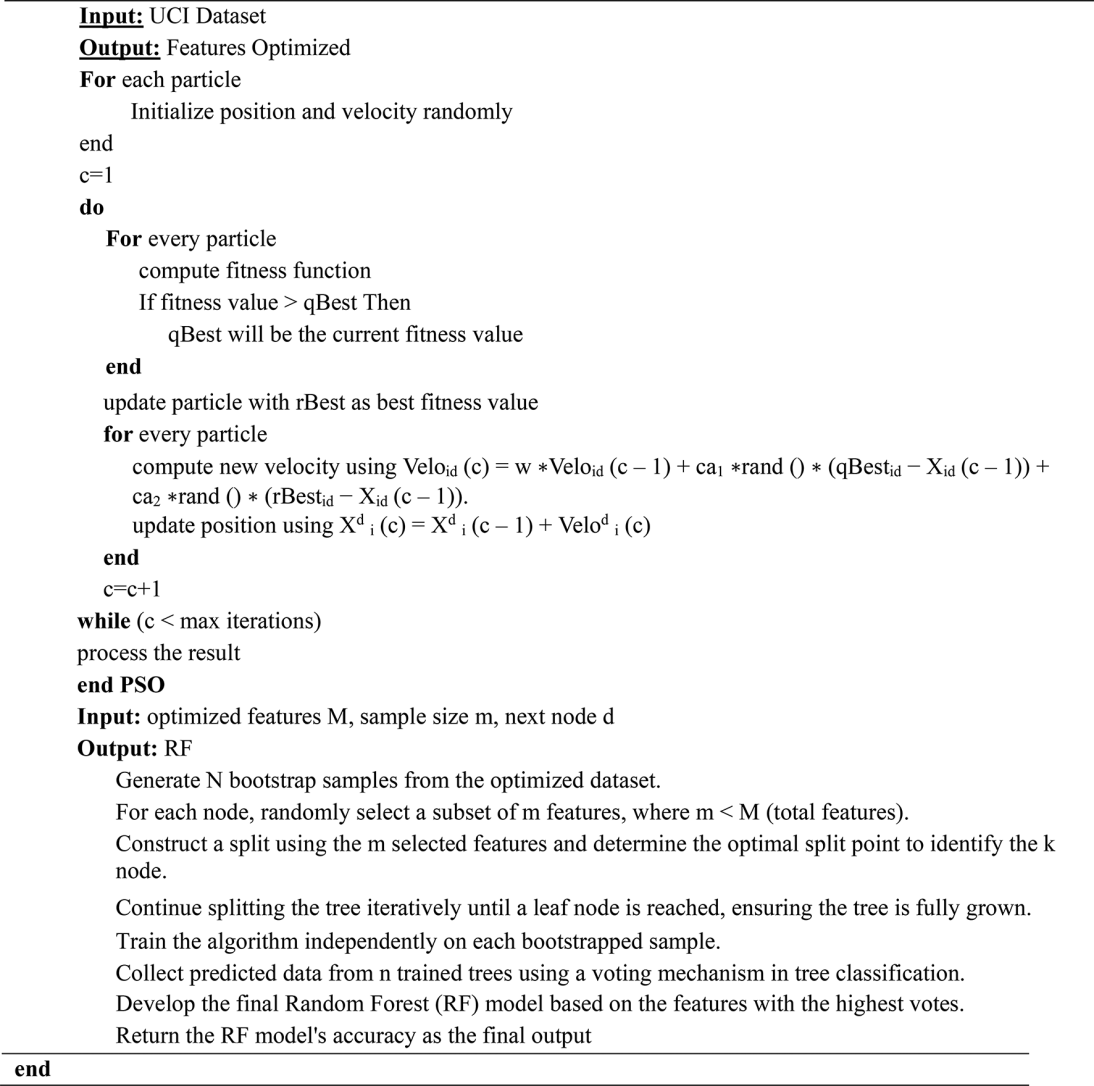
Pseudocode for Particle Swarm Optimization—(PSORF).

## Experiment results and analysis

### Performance measure

The effectiveness of the proposed method is evaluated based on the selected features and the accuracy of classification. The performance is assessed using key metrics, including accuracy, sensitivity, precision, and recall. The formulas used for calculating specificity, F-score, precision, recall, and accuracy are given in Table 3.

**Table 3:** Different evaluation criteria for assessing performance^37^.

Measure	Formula	Description
Precision	$\:\frac{TP}{TP+FP}$	The ratio of correctly predicted positive cases to the total number of actual positive cases
Recall	$\:\frac{TP}{TP+FN}$	The proportion of correctly identified positive cases relative to the total sample size
Specificity	$\:\frac{TN}{TN+FP}$	The number of correctly classified negative cases out of the total non-disease samples
Accuracy	$\:\frac{TP+TN}{TP+FP+FN+TN}$	The overall accuracy, representing the number of correct predictions out of all predictions made.
F-score	2*( $\:\frac{Precision\:*\:Recall}{Precision\:+Recall}$ )	The F-score is primarily utilized for addressing classification challenges, particularly when dealing with imbalanced class distributions.

### Design setup for experiment

The proposed model is evaluated using optimization techniques and machine learning models. The process begins with data collection, followed by preprocessing to manage any missing values. Next, the dataset undergoes optimization, and the selected features are utilized for classifying heart disease patients using a machine learning model. Various evaluation metrics are applied to assess performance. The experiment is conducted on Anaconda Jupyter Notebook 6.4.8, which includes built-in machine learning packages, and runs on an Intel(R) Core (TM) i7-7600U CPU @ 2.80GHz–2.90GHz.

### Feature selection results

This work investigates feature selection to optimize the accuracy of ML algorithms for heart disease prediction.  The authors employed the SelectKBest method with three filter methods: Chi-square, Mutual Information, and F-statistics. Each feature’s rank was determined by each filter, and an overall rank was calculated by combining these individual ranks mentioned in Table 4. The lowest-ranked features were removed, and the remaining features were fed into the classification algorithms. Using all features, Random Forest achieved the highest accuracy of 90.16% from Table 4. After applying feature selection, Random Forest experienced a slight accuracy drop. However, Logistic Regression performance improved, reaching 90.16% accuracy Table 6.

**Table 4:** Performance analysis for cleveland dataset before feature selection.

Algorithm	Accuracy (%)	Precision (%)	Recall (%)	F1-score (%)	Specificity (%)	Classification error rate (%)
LR	85.25	86	88	87	81	14.75
KNN	68.85	70	76	73	59	31.15
SVM	70.49	70	82	76	55	29.51
NB	85.25	84	91	87	78	14.75
RF with Grid Search	86.89	88	88	88	81	13.11
RF	90.16	89	94	91	85	9.84
XGB	81.97	83	85	84	77	18.03
DT	83.61	88	82	85	85	16.39
NN	83.61	80	94	86	43	16.39

**Table 5:** Cleveland dataset feature score table.

Cleveland dataset features	chi-square	MI	F-statistics	Total	overall rank
Age in years	4.501	0.001	3.452	7.954	11
Sex	1.465	2.802	5.525	9.792	9
CP-Chest pain	12.1	16.351	14.945	43.396	3
trestbps	2.866	4.232	1.383	8.481	10
chol	4.627	6.612	0.472	11.711	8
Fasting Blood sugar	0.039	1.315	0.051	1.405	13
Restecg—resting electrocardiographic	0.576	2.344	1.237	4.157	12
thalach	36.403	7.365	13.949	57.717	1
Exangexercise-induced angina	7.522	8.872	15.198	31.592	5
Old peak	14.042	15.663	14.684	44.389	2
Sl: slope	1.895	7.99	8.761	18.646	7
Ca	12.843	11.819	11.687	36.349	4
thal	1.12	14.365	8.655	24.14	6

**Table 6:** Accuracy of the heart disease dataset after feature selection along with the selected features

Dataset	classifier	Accuracy using all features	Accuracy of selected features using filter methods
Cleveland	RF	90.16	89(12)
	RFGS	86	87(8)
	NB	85.25	87(4)
	LR	85.25	90.16(4)
	XGB	81.97	87(6)

This section explores three metaheuristic algorithms GA ACO, PSO for feature selection in predicting heart disease.These algorithms aim to identify a subset of important features from the original dataset, potentially enhancing the accuracy of the prediction model. All three algorithms successfully reduced the initial 13 features to a smaller set. Genetic Algorithm and Particle Swarm Optimization selected 10 features each. Ant Colony Optimization selected 9 features shown in Table 7. This study will compare the performance of these optimized feature sets with existing models utilizing all 13 features. Each optimized feature set will be fed into a Random Forest classifier to assess its impact on prediction accuracy. The results will be presented, comparing the accuracy achieved by different feature selection methods and traditional approaches using all features. In medical predictions, accuracy is paramount. By identifying the most relevant features, these algorithms have the potential to improve the model’s ability to correctly diagnose heart disease, potentially leading to better patient outcomes.

The parameters considered for GAO are.

Mutation Rate: Varied (1000, 100, 5).

Crossover Rate: Implicitly 100%.

Crossover Operator: Single-Point Crossover.

Number of Generations: 50 and 100 in different runs.

Population Size: 20.

Stopping Criteria: Number of generations.

Selection Operator: Fitness-proportional selection from a randomly chosen subset of 5 individuals.

Selection Pressure: Implicit in the selection operator.

Fitness Function: Based on the inverse of the root mean squared error from a Random Forest Regressor.

The parameters considered for PSO are.

Features = 13.

C1 = 0.5 (Cognitive factor influencing individual particle movement).

C2 = 0.5 (Social factor guiding particle interaction within the swarm).

W = 0.9 (Inertia weight controlling the influence of previous velocity).

K = 30 (Number of top-performing particles or neighboring particles considered).

*P* = 2 (Parameter defining the search space dynamics).

Iterations = 1000.

The parameters considered for ACO are.

iteration = 100.

n_ants = 5 (Total number of ants in the system).

n_citys = 5 (Total number of cities or nodes to be visited).

e = 0.5  (Evaporation rate, determining pheromone decay over time).

alpha = 1 (pheromone influence factor, controlling the impact of pheromone trails).

beta = 2 (visibility factor, emphasizing the importance of heuristic information in decision-making).

Based on the parameters used by each optimization algorithm, the features are selected and are listed in Table 7.

**Table 7:** List of subsets of features selected based on metaheuristic techniques.

Metaheuristic technique	Number of features selected	Subset of features
Genetic Algorithm	10	Age, sex, cp., trestbps, chol, fbs, thalach, oldpeak, slope, thal
Particle Swarm Optimization	10	sex, cp., trestbps, chol, fbs, restecg, thalach, oldpeak, exang, thal
Ant Colony Optimization	9	Age, cp., trestbps, chol, fbs, restecg, thalach, exang thal

 RF algorithm is performed for the features selected by GA, ACO, PSO. Table 8 records the performance metrics achieved after the feature selection by metaheuristics. Random forest with Genetic algorithm achieves 92% accuracy, precision, and recall with 94.1%, specificity of 92.59%, and Area under the curve of 95%.

Random forest with PSO achieves 91.8% accuracy, 94% recall 91.43% precision, specificity 88.89%, and Area under curve 93%. Random forest with ACO achieves only 86.88% accuracy, 91.18% recall, 86.11% precision, and specificity with 81.48% and 92% area under the curve. From Table 8, it is clear that GAORF performs better compared with other techniques and achieved 92%. The same is visualized with the help of a bar graph shown in Fig. 5.

**Table 8:** The result of performance metrics for different optimization techniques along with classification algorithm in terms of percentage (%).

Performance metrics	RF	GAORF	PSORF	ACORF
Recall	93	93.1	94	91.18
Precision	88.88	93.1	91.43	86.11
Accuracy	90.16	92	91	86.88
Specificity	85.18	91.59	88.89	81.48
AUC	90	94	93	92

**Fig. 6:**
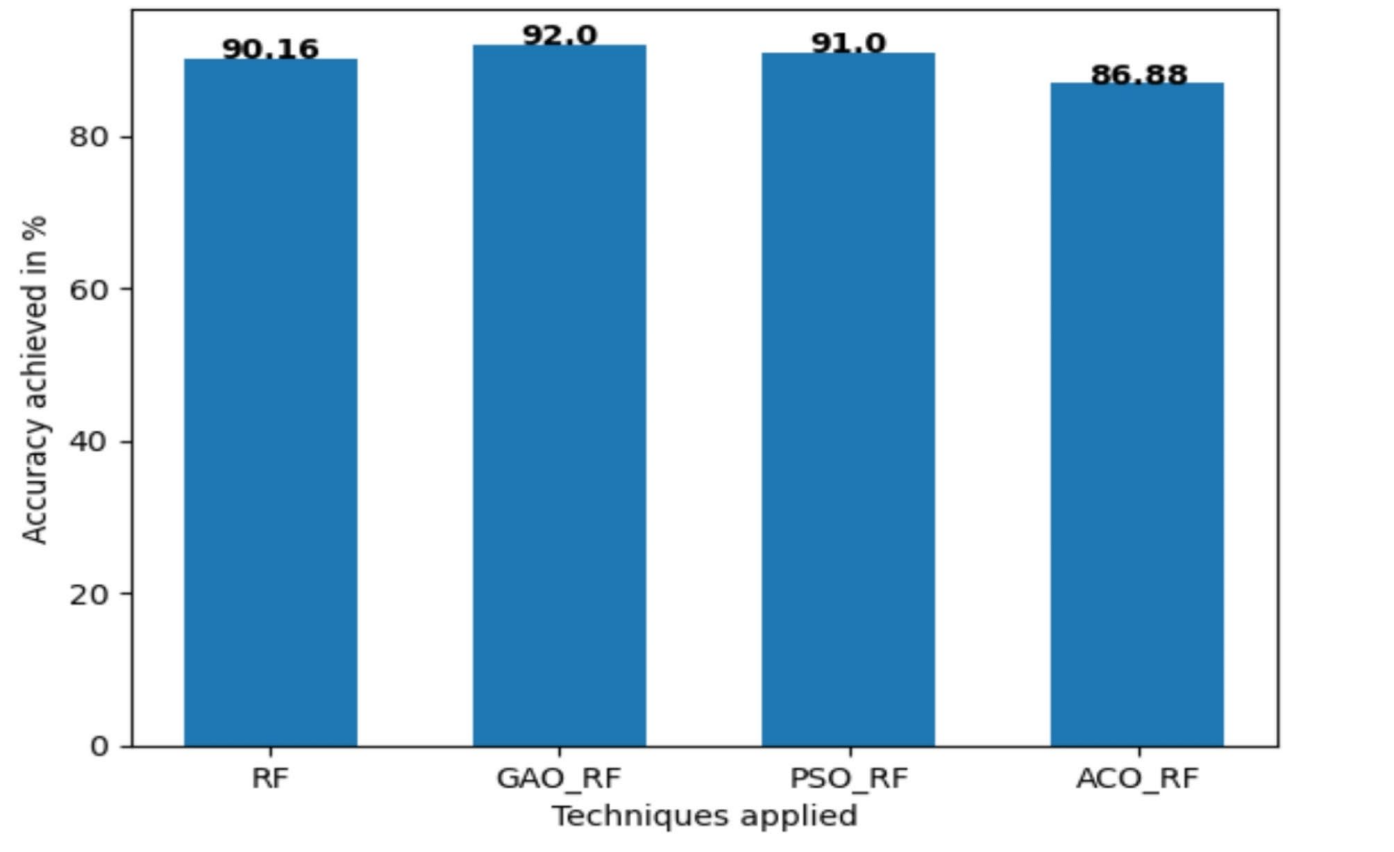
Comparing the accuracy of the optimization techniques along with RF for the Cleveland dataset.

GAORF Achieves Highest Accuracy in Heart Disease Prediction. Accuracy is a key metric in evaluating the effectiveness of prediction models. In this study, Fig. 6 visualizes the accuracy achieved by different algorithms applied to the Cleveland dataset. The proposed Genetic Algorithm-optimized Random Forest (GAORF) model outperformed all other techniques with a remarkable 92% accuracy. This result underscores the efficiency of GAORF in both feature selection and classification for heart disease prediction. Table 9 summarizes the performance of different machine learning algorithms. Random Forest (RF) alone achieved 90.16% accuracy, demonstrating its strong potential. Other methods like LR (85.25%), KNN (68.85%), SVM (70.49%), and NB (85.25%) displayed lower accuracy. Notably, Grid Search optimization slightly improved RF accuracy to 86.89%, but remained below GAORF.

**Table 9:** Comparing the performance of GAORF along with different classification algorithms.

Algorithm	Accuracy (%)	Precision (%)	Recall (%)	Specificity (%)	AUC (%)
Logistic Regression	85.25	86	88	81	91
KNN	68.85	70	76	59	73
SVM	70.49	70	82	55	81
NB	85.25	84	91	78	91
RF with Grid Search	86.89	88	88	81	90
RF	90.16	88.88	93	85.18	90
XGB	81.97	83	85	77	91
DT	83.61	88	82	85	84
GAORF	92	93.1	93.11	91.59	94

**Fig. 7:**
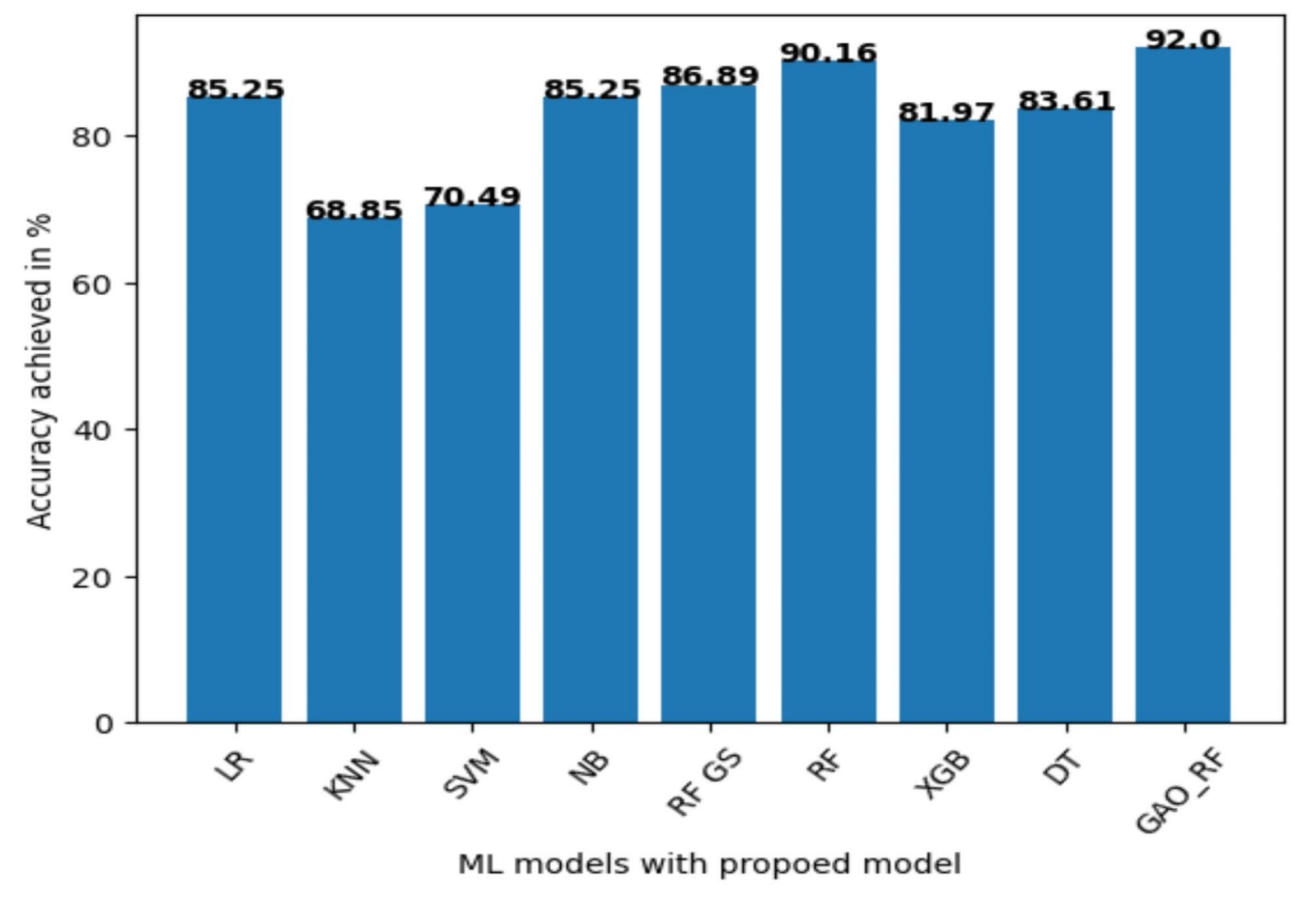
Comparing the accuracy of the proposed model along with the ML classification algorithm for the Cleveland dataset.

The different performance metrics for the proposed model GAORF are compared with varied Machine learning classification techniques. The performance is visualized with the help of Table 9. Figure 7 explains the accuracy variation between features selected using filter methods and optimization techniques. The line graph clearly explains that optimization techniques perform better compared with filter methods. Among the optimization techniques, GA achieved an accuracy of 92% in predicting heart disease. The performance metrics values are mentioned in Table 10.

**Table 10:** Comparing the accuracy of GAO + RF along with different classification algorithms before and after feature selection.

Dataset	classifier	Accuracy using all features	Accuracy of selected features using the filter method
Cleveland	RF	90.16	89(12)
	RFGS	86	87(8)
	NB	85.25	87(4)
	LR	85.25	90.16(4)
	XGB	81.97	87(6)
	GAORF	92	
	PSORF	91	
	ACORF	86.11	

### Discussion

While the proposed approach using GAORF achieved promising results for heart disease prediction, it’s important to acknowledge the inherent limitations associated with machine learning and metaheuristic optimization techniques. The following Table 11 highlights some key limitations of the methods explored in this paper.

**Table 11:** Proposed model strength and limitations.

Technique	Strengths	Limitations
GAORF	Handles non-linear complex problems	Can be computationally expensive. Tuning hyperparameters for GA can be challenging. May not be easily interpretable
PSORF	Relatively easy to implement. Well-suited for continuous optimization problems	May struggle with high-dimensional datasets (many features). Can get stuck in local optima (suboptimal solutions)
ACORF	Inspired by real-world ant behaviour for efficient searching	Performance can be sensitive to parameter settings. May converge slowly on complex problems
SelectKBest Feature Selection	Reduces computational cost and training time	May discard potentially useful features. Doesn’t consider feature interactions

**Table 12:** Proposed model vs existing models

Models	Accuracy
Proposed Model	92%
BABC–K-NN produced^16^	86.76%
GA + SVM^25^	87%
ACO + SVM^20^	87.77%
Best F measure^26^	90.50%
ACO^21^	85.21%
Modified Bee algorithm + RF^22^	85.20%
92HGDGWO^39^	80%
NB + FS^40^	84%
Fisher score algorithm + SVM^41^	81.91%
Hybrid (NB, BN, RF, and MP)^42^	85.45%
Modified GWO + LR^43^	86.91%
Modified GWO + KNN^43^	87.46%

 This study seeks to enhance heart disease prediction accuracy by integrating feature selection methods with machine learning algorithms. The work consists of 2 phases. Phase 1 is Feature Selection. The SelectKBest method with three filter methods (Chi-square, Mutual Information, and F-statistics) is employed to identify the most relevant features from the dataset. This reduced the initial feature set while potentially maintaining or even improving prediction accuracy. Initially, Random Forest performed best with 90.16% accuracy using all features. However, after applying feature selection, Logistic Regression showed better performance (90.16%). Phase 2 is applying Metaheuristic Optimization for Feature Selection. Observing the impact of feature selection on different algorithms, we explored metaheuristic techniques for further optimization. The study utilized GAO, PSO, and ACO to select even more impactful feature subsets. Each optimized feature set was then fed into a Random Forest classifier to assess its effectiveness. The combination of GAO and Random Forest (GAORF) achieved the highest accuracy of 92%, surpassing all other techniques. This demonstrates the effectiveness of GAORF in selecting optimal features for heart disease prediction. By combining metaheuristic optimization with machine learning, we were able to significantly improve prediction accuracy compared to using all features or basic feature selection methods. The existing model with the proposed model comparison is shown in Table 12.

## Conclusion

This research proposes a hybrid approach for predicting heart disease, focusing on maximizing accuracy and surpassing existing methods. An efficient machine learning model along with metaheuristic optimization techniques is applied to the heart disease dataset to enhance the accuracy in predicting the disease. The model is applied to the Cleveland Heart dataset. The optimization techniques namely GAO, PSO, and ACO are compared with the classification algorithms of machine learning. The model performed before and after optimization. Also, the model is compared with SelectKBest filter methods namely Chi-square, Mutual information, and F-statistics. The accuracy achieved by selecting features based on the overall rank of features is analyzed with the metaheuristic techniques. The output of the experiment explains that the GAORF performed better for the dataset considered. The proposed model achieved higher accuracy which is the maximum among other classification techniques. This study is limited to heart disease and its dataset. Future work can be done with other metaheuristic techniques and also different datasets available for predicting heart disease. The same methods can be applied to diabetes prediction and cancer prediction at the initial level.

## Data Availability

The datasets generated and/or analyzed during the current study are available in the UCI repository and can be accessed from (https://archive.ics.uci.edu/dataset/45/heart+disease).
